# Bibliographical

**Published:** 1882-12

**Authors:** 


### BIBLIOGRAPHICAL.

"THE DISEASES OF THE LIVER, with and without Jaundice, with the special application of Physiological Chemistry to their Diagnosis and treatment ; by George Harley, M. D., F. R. S., etc. Philadelphia : P. Blakiston, Son & Co., 1883." Pages 751 ; cloth, price $5 Prof. Harley is well known in England and America as a scientist, who has contributed largely to medical literature. His treatise on jaundice written some twenty years ago, received considerable favor. Add these long years of experience and scientific investigation of this organ and we naturally expect to find just such a book as lies before us. It treats specially of the physiology of the liver and its concomitant diseases. The chemistry and physics, also the etiology of jaundice are ably treated. Biliousness and jaundice receives minute detail. In fact the work treats carefully all the diseases of the liver, gall-bladder, and their diagnosis and prognosis. The publishers, too, have done their part well. Two colored lithographs and a large number of wood cuts adorn this admirable book.

"SORE THROAT ; its Nature, Varieties and Treatment ; including the connection between affections of the throat and other diseases ; by Prosser James, M. D.. Physician to the Hospital for Diseases of the Throat and Chest ; Fourth Edition, England ; with Colored Plates and Wood Engravings. Philadelphia : P. Blakiston, Son & Co." Pages 318 ; paper 75cts. This little volume merits what its title page sets forth. Now is the season in which this "handbook for physicians" should be popular.

Dental Metallurgy ; A Manual for the use of dental students, by Chas. J. Essig, M. D., D. D. S., Professor of Dentistry and Metallurgy in the Dental Department of the University of Pa., (Philadelphia) : The S. S. White Dental Mfg. Co." Pages, 253. Prof. Essig has done the student of mechanical dentistry a valuable service.

Pamphlets Received.—We regret that time will not permit us to give these interesting and valuable papers individual notice, further than the titles and the authors' names, viz: "Oleates and Oleo-Palmitates in Skin Diseases," "The Therapeutic Action of Potassium Chlorate," "The Treatment of Syphills with Subcutaneous Sublimate Injections, by Jno. V. Shoemaker, A. M., M. D., of Phila., " Use of the Ecraseur for Curing Deep-Seated Fistula in Ano, by J. M. F. Gaston, of Campinas, Brazil.' "Some Points on the Administration of Anæsthetics, by George H. Rohé, M. D., Prof. of Hygiene and Clinical Dermatology, College of Physicians and Surgeons, Baltimore." " Nervous Force ; its Origin and Physiology," by W. C. Barrett, M.D., D.D.S., of Buffalo, N.Y. "The Trance State in Inebriety ; its Medico Legal Relations, by T. D. Crothers, M.D., Superintendent Walnut Lodge, Hartford Conn., with an Introduction on the Nature and Character of the Trance State, by Geo. M. Beard, M. D., of New York City. " Conjoint Session of the N. Carolina Board of Health and the Medical Society of N. Carolina, held in Concord, May 10th, 1882." "Transactions Mississippi State Medical Association, Oxford, April, 1882." These two society reports reflect credit upon the medical fraternity of the states of North Carolina and Mississippi.

